# Trophic redundancy and predator size class structure drive differences in kelp forest ecosystem dynamics

**DOI:** 10.1002/ecy.2993

**Published:** 2020-02-28

**Authors:** Jacob H. Eisaguirre, Joseph M. Eisaguirre, Kathryn Davis, Peter M. Carlson, Steven D. Gaines, Jennifer E. Caselle

**Affiliations:** ^1^ Department of Environmental Studies University of California Santa Barbara California 93106 USA; ^2^ Marine Science Institute University of California Santa Barbara California 93106 USA; ^3^ Department of Biology & Wildlife University of Alaska Fairbanks Fairbanks Alaska 99775 USA; ^4^ Department of Mathematics & Statistics University of Alaska Fairbanks Fairbanks Alaska 99775 USA; ^5^ Bren School of Environmental Science and Management University of California Santa Barbara California 93106 USA

**Keywords:** California sheephead, California spiny lobster, kelp forest, linear mixed effects models, managing for resilience, phase shifts, predator guilds, sea star wasting disease, size class structure, sunflower sea star, trophic redundancy, urchin barrens

## Abstract

Ecosystems are changing at alarming rates because of climate change and a wide variety of other anthropogenic stressors. These stressors have the potential to cause phase shifts to less productive ecosystems. A major challenge for ecologists is to identify ecosystem attributes that enhance resilience and can buffer systems from shifts to less desirable alternative states. In this study, we used the Northern Channel Islands, California, as a model kelp forest ecosystem that had been perturbed from the loss of an important sea star predator due to a sea star wasting disease. To determine the mechanisms that prevent phase shifts from productive kelp forests to less productive urchin barrens, we compared pre‐ and postdisease predator assemblages as predictors of purple urchin densities. We found that prior to the onset of the disease outbreak, the sunflower sea star exerted strong predation pressures and was able to suppress purple urchin populations effectively. After the disease outbreak, which functionally extirpated the sunflower star, we found that the ecosystem response—urchin and algal abundances—depended on the abundance and/or size of remaining predator species. Inside Marine Protected Areas (MPAs), the large numbers and sizes of other urchin predators suppressed purple urchin populations resulting in kelp and understory algal growth. Outside of the MPAs, where these alternative urchin predators are fished, less abundant, and smaller, urchin populations grew dramatically in the absence of sunflower stars resulting in less kelp at these locations. Our results demonstrate that protected trophic redundancy inside MPAs creates a net of stability that could limit kelp forest ecosystem phase shifts to less desirable, alternative states when perturbed. This highlights the importance of harboring diversity and managing predator guilds.

## Introduction

Keystone species often play important roles in sustaining the functioning of ecosystems and can be found occupying niches at various trophic levels (Power et al. 1996). Loss of keystone species at higher trophic levels can ultimately affect lower trophic levels through cascading forces that alter ecosystem structure and function (Paine 1966, Power et al. 1996, Estes et al. 2004). In both terrestrial and marine systems, overharvesting and climate change have driven dramatic changes in the populations of foundational and keystone species, thus resulting in abrupt and sudden phase shifts (Estes and Palmisano 1974, Daskalov et al. 2007, DeYoung et al. 2008, Ling et al. 2015, McCary et al. 2016). This trend has led to great concern about losses of biodiversity and valuable ecosystem services and spurs the need to better understand and manage for ecosystem traits that confer resilience to communities and ecosystems (Folke et al. 2004).

There is a growing body of evidence that high‐diversity systems may have enhanced ecosystem stability and resilience potential. These benefits can arise due to the number and strength of species interactions (McCann et al. 1998) and redundancy in traits and trophic roles (Menge 1983, Peterson et al. 1998, Steneck et al. 2002, Snyder et al. 2008). Ecosystems that exhibit redundancy in important functions have been shown to be more resistant to the cascading effects of disturbance and extinction (Borrvall et al. 2000, Finke and Denno 2004, Dunne and Williams 2009, Sanders et al. 2018). However, much of our understanding of the relationships between diversity, trophic redundancy, and ecosystem resilience comes from theoretical models, controlled experiments, and hypotheses derived from large‐scale correlative observations. There are few clear demonstrations of this concept in complex natural systems (but see Burt et al. 2018, McLean et al. 2019).

The Northern Channel Islands (NCI), in the Southern California Bight, provide a unique setting to explore how trophic redundancy and predator diversity strengthen resilience in kelp forest ecosystems by examining patterns occurring at the intersection of several features: historical overfishing, a historically diverse predator guild, the establishment of a large network of marine protected areas (MPAs), and a recent disturbance event. Here we explore how the disturbance, the loss of a single predator due to a widespread disease epidemic, affects populations of its preferred prey, a barrens‐forming, herbivorous sea urchin. The presence of two additional fished predators in the system along with spatial variation in fishing pressure due to the MPAs allows us to test whether functional redundancy in the predator guild has buffered the system from the cascading effects of the loss of an important predator.

Kelp forests are iconic and highly dynamic coastal ecosystems that provide a multitude of ecological functions and valuable ecosystem services (summarized in Krumhansl et al. 2016). One of the major threats to kelp forests is degradation due to overgrazing by herbivorous sea urchins, which can rapidly shift a healthy kelp forest to an urchin barren when present in high densities (Harrold and Reed 1985, Steneck et al. 2002, Filbee‐Dexter and Scheibling 2014, Ling et al. 2015). Once established, urchins in barrens may prevent the recolonization of kelps by consumption of spores and recruits and thereby stabilize the system in a degraded state (Chapman 1981). In a global analysis, Ling et al. (2015) found that such discontinuous phase shifts with hysteresis effects have been recorded in a number of macroalgae‐dominated ecosystems. Notably, across studies it was found that the minimum urchin density to initiate phase shifts from healthy kelp forests to unproductive urchin barrens occurs at ~11–14 urchins/m^2^ but that once barrens are established urchin densities have to be reduced by an order of magnitude for kelp to recover (Filbee‐Dexter and Scheibling 2014, Ling et al. 2015, Dunn and Hovel 2019). Phase shifts with hysteresis can often have catastrophic effects on fisheries and other ecosystem services when systems are shifted into unproductive alternative states. In such situations, it is important for wildlife managers to manage for resilience, where possible, through understanding the thresholds and feedback mechanism that can foster ecosystem stability and prevent phase shifts.

Kelp forests are complex, disturbance‐driven systems that respond to a wide variety of top‐down and bottom‐up controls (Dayton 1985, Steneck et al. 2002, Reed et al. 2011, Byrnes et al. 2013, Pérez‐Matus et al. 2017), however, in many kelp systems there is good evidence for strong cascading effects of urchin predators on foundational kelps through their control of urchin populations (Estes and Palmisano 1974, Shears and Babcock 2002, Behrens and Lafferty 2004, Hamilton and Caselle 2015). Diverse suites of predatory species can further increase top‐down control of urchin populations by differentially targeting various size classes (Burt et al. 2018), and by modifying urchin behaviors (Byrnes et al. 2006), ultimately stabilizing and enhancing ecosystem resilience through the increased top‐down control (Borrvall et al. 2000, Finke and Denno 2004, Dunne and Williams 2009, Sanders et al. 2018).

**Figure 1:**
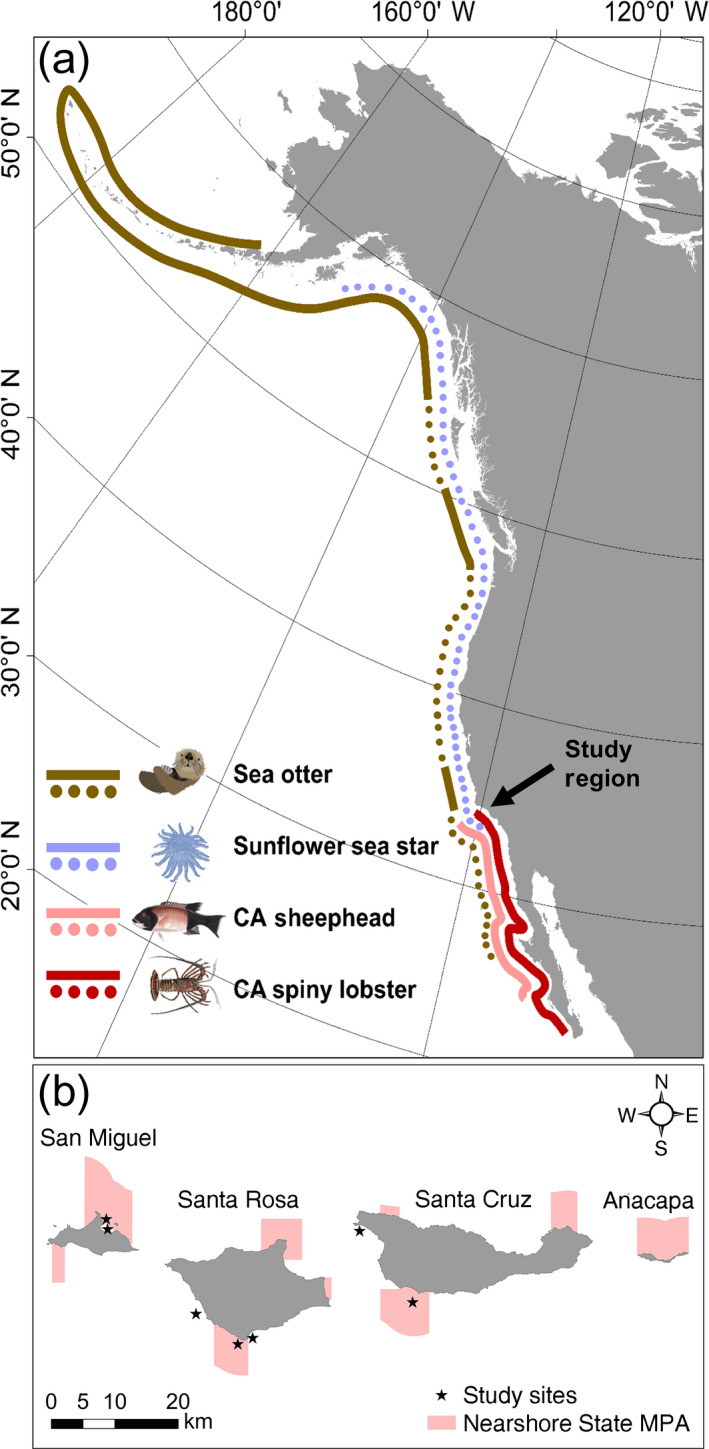
(a) Approximate current (solid lines) and historical (dotted lines) distribution along the Pacific coast of major predators of purple and red sea urchins. (Estes and Duggins 1995, Pringle 2011, Bodkin 2015, Pondella et al. 2015, Fishbase.org). California sheephead and spiny lobster historical and current ranges are the same, so only current range is shown. Map of North America is projected in North America Albers Equal Area Conic for display purposes. (b) Study sites and Nearshore State Marine Protected Areas (MPAs) located at the Northern Channel Islands (NCI).

The urchin predator guild along the west coast of North America is made up of four dominant species; sea otters (*Enhydra lutris*), sunflower sea stars (*Pycnopodia helianthoides*), California (CA) sheephead (*Semicossyphus pulcher*, a labrid fish), and California (CA) spiny lobsters (*Panulirus interruptus*) (Fig. 1a). These four species vary both temporally and spatially in their distributions across the region. Sunflower sea stars, until recently, occurred from southern Alaska to the Southern California Bight (Estes and Duggins 1995, Pondella et al. 2015) and were important urchin predators across this region (Moitoza and Phillips 1979, Duggins 1983, Schultz et al. 2016). Recently, a sea star wasting disease epidemic has affected more than 20 sea star species ranging from Alaska to Mexico (Hewson et al. 2018). Sunflower sea stars were particularly susceptible to the effects of the disease, and by 2014 they were functionally extirpated from most of their historic range (Schultz et al. 2016, Harvell 2019). Sea otters*,* a well‐known keystone predator of urchins in North Pacific kelp forests, were historically present along much of Northeast Pacific Coast until populations were decimated by intense overharvesting for the fur trade in the early 1800s (Estes and Palmisano 1974, Estes et al. 2004). Today, sea otters only persist in high densities in a fraction of their historic range (Bodkin 2015). In British Columbia, where otters and sunflower sea stars historically co‐occurred, Burt et al. (2018) showed that sea otters and sunflower sea stars display niche complementarity in their predation on sea urchins, resulting in strong top‐down control when both predators were present. Using natural variability in the presence/absence of sea otters, they showed that after the extirpation of sunflower sea stars from disease, urchin densities increased and kelp declined, but that this result was much stronger in the absence of sea otters. This demonstrates that trophic redundancy in urchin predators had allowed kelp forests in the North Pacific to persist after the loss of a single top predator.

The Northern Channel Islands (NCI) are outside of the present‐day range of sea otters, but host two additional major urchin predator species, which, along with sunflower sea stars, comprised a diverse predator guild that likely fostered robust kelp forests in the region (Steneck et al. 2002). CA sheephead and CA spiny lobsters are both generalist predators that prey upon purple urchins (*Stronglyocentrotus purpuratus*), with both species increasing their effectiveness as urchin predators with increasing body size (Behrens and Lafferty 2004, Eurich et al. 2014, Hamilton et al. 2014, Hamilton and Caselle 2015, Selden et al. 2017). The waters surrounding the western NCI, where the present study was conducted, have a mosaic of protection status through a network of MPAs that was established in 2003. Within the MPAs, the fished predators (CA sheephead and CA spiny lobsters) are released from commercial and sportfishing pressures. Their numbers and sizes are significantly greater in the MPAs relative to nearby fished areas (Lafferty 2004, Hamilton et al. 2010a *a*, Caselle et al. 2015, Selden et al. 2017).

Using the extirpation of the unfished predator—the sunflower sea star—and the presence of a mosaic of protected areas for the other two predators as a “natural” experiment, we test the effects of predator identity, abundance, and size structure on the density of purple urchins and the consequent patterns of kelp abundance. We utilize data from a long‐term monitoring study that spans the periods before and after the loss of the sunflower sea star. Specifically, we ask how MPAs might facilitate trophic redundancy through targeted predator protection and influence patterns of size‐specific foraging on purple urchins. We then ask how purple urchin abundance affects both canopy‐forming giant kelp and understory kelp. We conduct this work in the western portion of the Northern Channel Islands in order to constrain variability in some of the other drivers of kelp abundance including productivity, upwelling, and wave exposure. We hypothesize that in places with greater redundancy of predators, trophic effects will be stronger, resulting in fewer urchins and more kelp.

## Methods

### Study sites

We conducted the study at seven sites across three islands at the western NCI (Fig. 1b). Historically, sunflower sea stars were prevalent in the colder waters of the western islands and uncommon in the eastern NCI (Bonaviri et al. 2017), so we restricted our study area to the western portion of the region. Restricting the study area to the western islands also allowed us to better control for environmental conditions (e.g., sea temperature, productivity; see Hamilton et al. 2010b *b*, Pondella et al. 2015, 2019). All MPAs used for this study are no‐take state marine reserves where all forms of fishing are banned.

### Community surveys

Kelp forest community surveys have been conducted in this region since 1999 by the Partnership for Interdisciplinary Studies of Coastal Ocean (PISCO). Surveys are conducted annually in rocky reefs/kelp forests in MPAs and reference areas (sites where fishing is permitted) at depths less than 25 m. Fish surveys are conducted at three levels of the water column: benthic, midwater, and canopy (if present) between August and October. Twenty‐four fish transects (30 × 2 × 2 m) are conducted at each site in order to characterize fish community structure. All fishes (except very small‐bodied and cryptic species) are identified and sized to the nearest centimeter by the diver. Fish surveys are stratified into four depth zones, targeting 5‐, 10‐, 15‐, and 20‐m bottom depth, although the depth of surveys varies slightly per site depending on reef topography. In addition, 12 “benthic” transects (30 × 2 m) are surveyed at each site between June and August to quantify densities of invertebrates and macroalgae. Benthic surveys are stratified into three depth zones (approximately 5‐, 10‐, and 15‐m depth). Giant kelp (*Macrocystis pyrifera*) individuals greater than 1‐m in height are counted and stipes are enumerated per individual and later summed at the transect level for analysis. Understory kelps, *Eisenia arborea*, *Pterygophora californica*, *Laminaria farlowii*, *Laminaria setchellii* greater than 30 cm in height and the common fucoid *Stephanocystis osmundacea* greater than 6 cm across are also counted along benthic transects and were later pooled for analysis. Sea stars and purple urchins greater than 2.5 cm in diameter and lobsters of all sizes are also counted. At high densities, benthic organisms are subsampled within 10‐m segments of the transect using a variable area subsampling methodology, with the exception of giant kelp, which is not subsampled. Benthic data (urchin, sea star, lobster, understory algae, and kelp densities) were analyzed at the transect level. Giant kelp individual density and understory algal density were combined to create a total macroalgal density at a per‐transect level for analysis. Fish abundance and size data were averaged at the site level to account for the high mobility of the CA sheephead relative to the invertebrates (Topping et al. 2006).

### Sea surface temperature

Sea surface temperature (SST) was provided by the Santa Barbara Channel Marine Biodiversity Observation Network (SBC MBON). SBC MBON acquires SST with the Multiscale Ultra‐high Resolution Sea Surface Temperature satellite (MUR SST) in daily incremental grids at each PISCO survey site. SST data were then converted into annual site‐level means.

### Analyses

We developed and fit a candidate set of generalized linear mixed effect models (GLMMs) of purple urchin densities using the R package lme4 (Bates et al. 2015, R Development Core Team 2018). Models were fit to the natural logarithm of the response (purple urchin density) using the identity link. We also fit a set of models to kelp densities, *Macrocystis pyrifera*,* Eisenia arborea*, *Pterygophora californica*, *Laminaria farlowii*, *Laminaria setchellii*,* and Stephanocystis osmundacea* (we include this fucoid algae with kelps because of its large, habitat‐forming growth form)*,* to ensure our data support known trophic relationships between macroalgae and urchins; these results are presented in the electronic supplementary material (ESM). Because we expected the processes driving urchin density to vary pre‐ and post‐extirpation of sunflower sea stars, we fit and ranked candidate models separately to these periods, which corresponded to 2010–2012 and 2014–2017, respectively. Data from 2013 were insufficient because few sites were sampled and therefore were not included. The period of interest began in 2010, prior to the onset of the widespread sea star disease. By 2014, sunflower sea stars were absent from our study sites (with no observed/expected recovery to date). Site‐specific intercepts (i.e., random effect of site) were included in the models to account for variation among sites.

We suspected that SST would explain much of the predictable variation in macroalgal density and purple urchin density across the NCI (Ohgaki et al. 2018), so we did not include a random effect of island, as there is a strong east–west temperature gradient across the islands. SST can also vary interannually due to the El Niño–Southern Oscillation (ENSO). We included transect depth in the models, instead of a random effect of transect, in order to explore the influence of depth on macroalgal and purple urchin abundance. We predict that macroalgal and purple urchin densities may vary with depth due to influences of wave surge, available sunlight, temperature, and algal distributions.

We ranked candidate models of macroalgal and urchin density with Akaike’s information criterion corrected for small sample sizes (AICc; Burnham and Anderson 2004). To illustrate the effects of different predictors on macroalgal and urchin density, we generated predictions from the fitted models. Prediction intervals were estimated with the R package merTools (Knowles and Frederick 2018). Homoscedastic variance, linearity, and the presence of outliers were checked by visual inspection of residual plots for the top‐ranking models.

### Candidate models

Prior to analyses, we generated several hypotheses for trophic drivers of macroalgal densities (Reed et al. 2011) and for why purple urchin densities may differ in MPAs and reference areas. We then constructed multiple models based on these hypotheses, including predator assemblages and abiotic factors (temperature and depth) to test their effects on controlling macroalgal and purple urchin populations pre‐ and postextirpation of sunflower sea stars (Appendix S1: Tables S1–S3). Models used to predict macroalgal densities included variations of temperature, depth, and purple urchin densities as additive variables.

**Figure 2:**
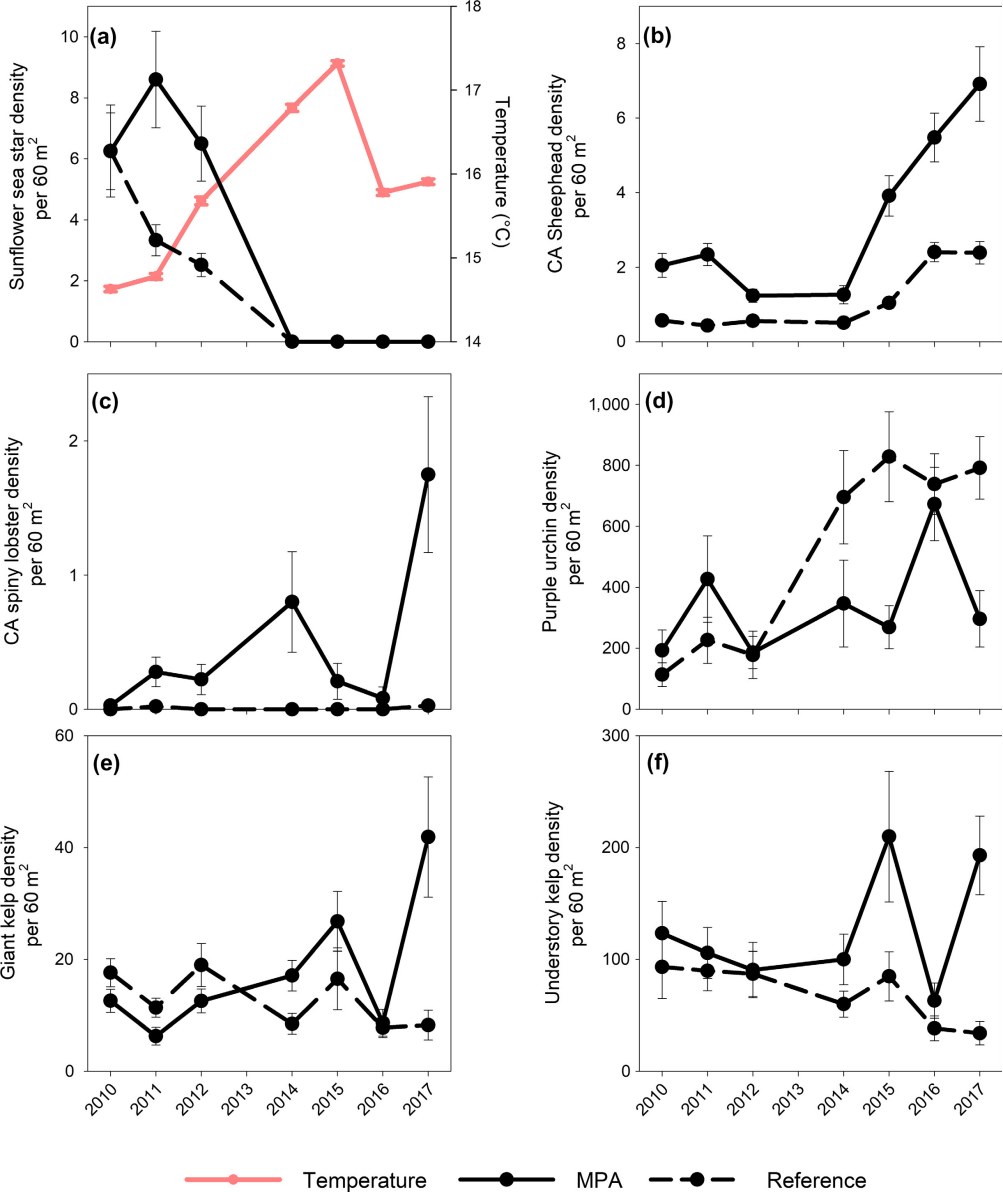
Temporal patterns of key members of the kelp forest community and sea surface temperature from 2010 to 2017. Solid lines are marine protected areas (MPA) sites, dashed lines are reference sites. All species data are mean density (per 60 m^2^) ± 1 standard error. (a) Sunflower sea star and temperature (°C), (b) California (CA) sheephead, (c) California spiny lobster, (d) purple urchin, (e) giant kelp, and (f) understory kelp.

Red urchins (*Mesocentrotus franciscanus*) were not included in the models because the predators in our study region show preference for purple urchins, likely due to stronger defenses due to longer spines of red urchins (Moitoza and Phillips 1979, Tegner and Levin 1983). Lobster density was not included in purple urchin models due to colinearity with CA sheephead (*r* = 0.78; Fig. 2b, c) and because very few (nearly zero) lobster were detected in the fished reference areas (Fig. 2c). Temperature and depth were additive variables in all models, which, as noted above, helped account for variation in island biogeography and variation among transects. We included an interaction between sunflower sea stars and protection status in purple urchin models, because we expected their effect on urchin density to vary with the level of protection due to interspecific competition with the other two predators (i.e., CA sheephead, CA lobster) both of which have been shown to benefit from MPA protections (Lafferty 2004, Hamilton and Caselle 2015). We also expected size‐specific effects of CA sheephead due to gape limitation and size‐specific predation rates (Selden et al. 2017), so the effect of CA sheephead was included in purple urchin models as the interaction between mean density and mean length per site. Collinearity between the predictors included in the final set of candidate models was checked with variance inflation factors (VIFs), which were all <2.4 (Appendix S1: Tables S4‐S5).

## Results

Sunflower sea stars were completely extirpated by disease from the rocky reef/kelp forest communities in the western NCI by 2014, which corresponded with strong temperature increases associated with an ENSO event (Fig. 2a). Prior to the extirpation of sunflower sea stars, the magnitude of difference between CA sheephead density inside and outside of reserves was relatively stable. Postextirpation, those differences increased (Fig. 2b). CA spiny lobster densities remained extremely low in the western NCI, pre‐ and postextirpation in both the reference and MPAs; however, CA spiny lobster densities were slightly higher in the MPAs and continued to increase postextirpation of sunflower sea stars (Fig. 2c). In general, purple urchin densities were similar in the MPAs and reference sites prior to the extirpation of sunflower sea stars, but coinciding with the loss of sea stars, purple urchin densities increased dramatically in the reference areas (Fig. 2d). Inside MPAs purple urchin density were variable following the loss of the sea stars (Fig. 2d). Both kelp stipe and understory algal densities varied over time (Fig. 2e, f). However, in the reference areas, algae showed a steady decline resulting in very low density, especially in recent years. Inside the MPAs, despite interannual variation, kelp and understory algal densities were greater than the reference areas (Fig. 2e, f).

We note that although our field observations of urchin barrens formation following the loss of sunflower sea stars indicated that barrens became more common and widespread, particularly in the reference areas (motivating the current analysis), we did not necessarily observe wholesale, site‐level phase shifts at all sites such as reported elsewhere in the world. Reference‐area sites showed overall declines in canopy forming and understory kelps, but retained some patches of kelp in places (Fig. 2e, f). Similarly, we did observe some high‐density patches of urchins developing in the MPAs but to a lesser extent than in the reference areas and without the resulting site‐level declines in macroalgae (Fig. 2d–f). When analyzed at finer spatial scales, at the transect level, we see supporting evidence for small‐scale patch dynamics, with barrens being more common in the reference areas than in the MPAs after the extirpation of sunflower sea stars, but with a high degree of variability in the abundance of urchins and algae within and across sites (Appendix S1: Fig. S1).

**Figure 3:**
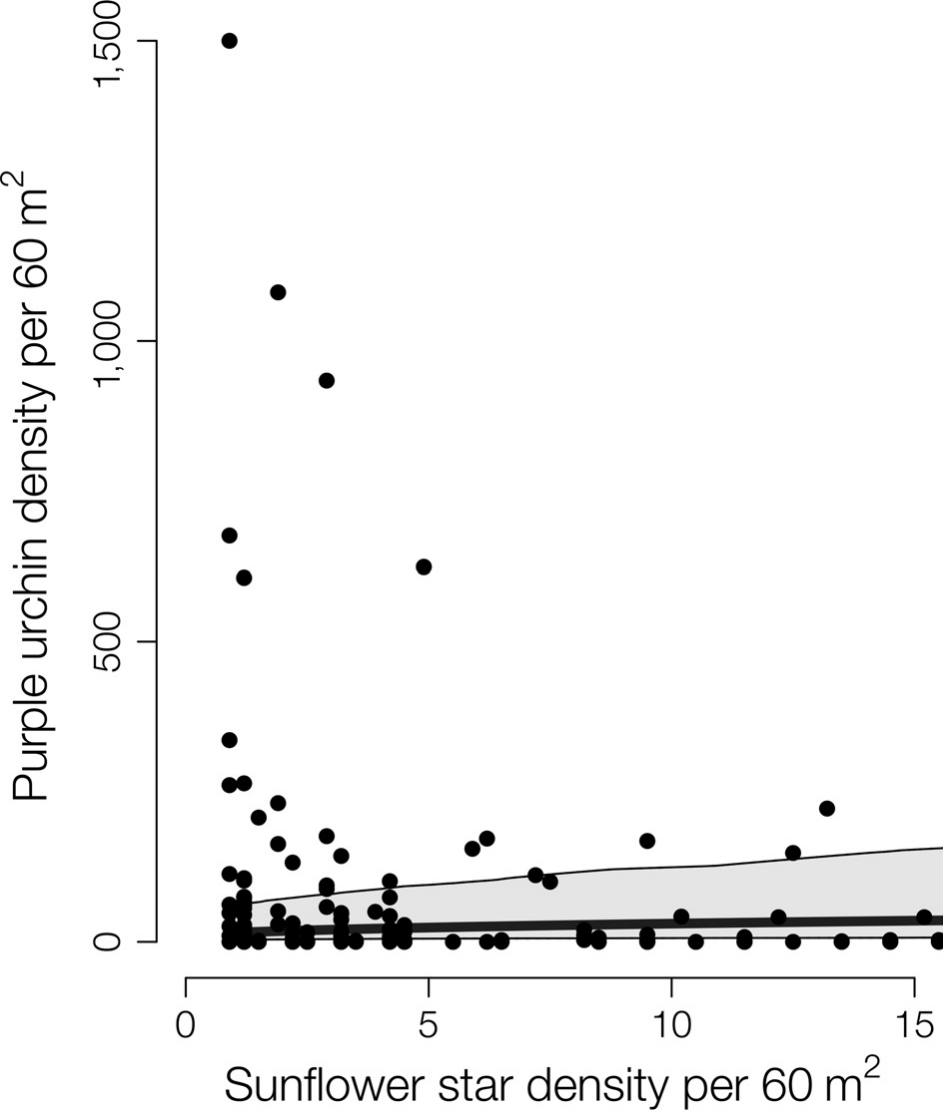
Empirical data (filled circles) and model prediction (line) of purple urchin densities (per 60 m^2^) with temperature, depth, and sunflower sea star densities (per 60 m^2^) as predictors with 95% prediction intervals both inside marine protected areas (MPAs) and reference sites at the Western Northern Channel Islands.

Prior to the extirpation of sunflower sea stars, the top model (lowest AICc and highest AICc weight [0.51]) for purple urchin density included fixed effects of temperature, depth, and sunflower sea star densities (Appendix S1: Table S1a) and a random effect of site (variance partition coefficient VPC = 0.615). Examining residual plots indicated linearity between the predictors and response, and homoscedastic variance; we also did not find any outliers or overly influential points (Appendix S1: Fig. S2). Interestingly, model predictions indicate purple urchin populations both in the MPAs, and reference areas were low when sunflower sea stars were present at any density (Fig. 3).

**Figure 4:**
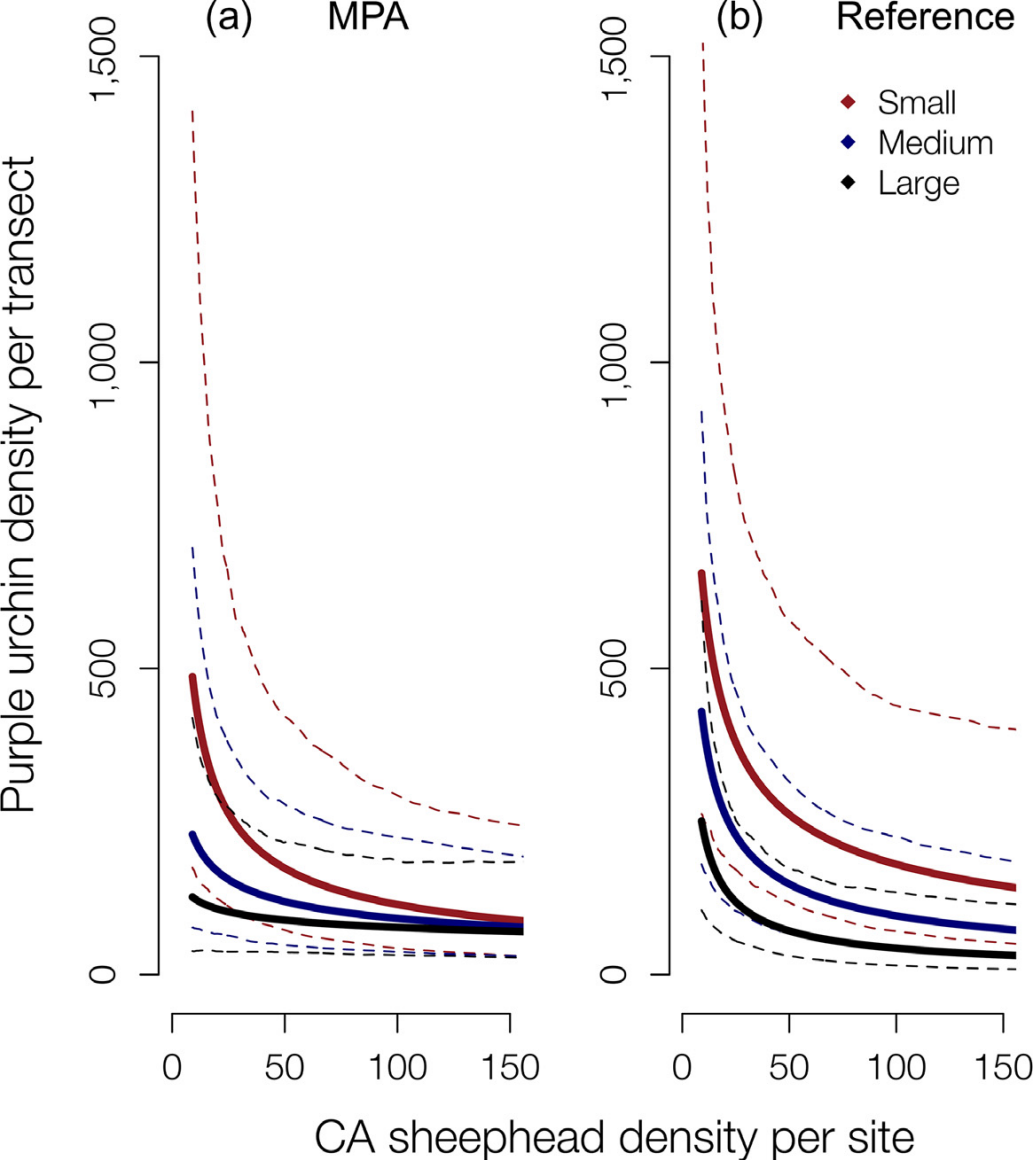
Predictions of purple urchin densities (per 60 m^2^) from model containing temperature, depth, California (CA) sheephead abundance (per site [1,440 m^2^]), and CA sheephead total length (TL) as predictors with 95% prediction intervals (dotted lines) (a) inside MPAs and (b) at reference sites at the Western Northern Channel Islands. CA sheephead lines indicate small‐sized CA sheephead = 24.57 cm TL (red line; quartile 1 of CA sheephead TL), medium = 31.50 cm TL (blue line; quartile 2 of CA sheephead TL), large = 38.15 cm TL (black line; quartile 3 of CA sheephead TL).

Postextirpation of sunflower sea stars, the top model (lowest AICc and highest AICc weight [0.99]) included fixed effects of temperature, depth, CA sheephead abundance, CA sheephead total length, and protection status (Appendix S1: Table S2a) and a random effect of site (VPC = 0.158). Again, examining residual plots indicated linearity between the predictors and response, homoscedastic variance, and no outliers nor overly influential points (Appendix S1: Fig. S3). Empirically measured mean CA sheephead total length (TL) and abundance were larger inside the MPAs ($\bar{X}$_TL_ = 33.45 cm [S_TL_ = 9.03 cm], $\bar{X}$_Abundance_ = 98.35 [SD_Abundance_ = 77.06]) compared to reference areas ($\bar{X}$_TL_ = 30.34 cm [SD_TL_ = 7.89 cm], $\bar{X}$_Abundance_ = 42.91 (SD_Abundance_ = 34.81), indicating that the urchin predator demographics are different, with larger more numerous individuals within the MPAs. We plotted the predicted urchin densities from this model against CA sheephead density for three different size classes of CA sheephead (small = 24.56 cm, medium = 31.50 cm, and large = 38.15 cm; sizes were calculated based on the first, second, and third quartiles of CA sheephead sizes in the empirical field data set). Predictions from the fitted model suggest effects of CA sheephead abundance and size on purple urchin densities are stronger in MPAs (Fig. 4a). Large CA sheephead are more effective at suppressing purple urchins at lower abundances than other size classes, yet even smaller CA sheephead can also suppress purple urchin populations when present at high abundances (Fig. 4a). Outside of MPAs, the effects that CA sheephead size and abundance have on purple urchin populations are similar to the effects in MPAs, yet of smaller magnitude (Fig. 4b).

Filbee‐Dexter and Scheibling (2014) summarized the threshold urchin densities needed to reverse phase shifts from barrens to kelp in California using data from Dean et al. (1984) and Dayton et al. (1992). The threshold densities were 2–3 individual urchins/m^2^. We asked how many CA sheephead of different size classes (TL) would be required to produce an urchin density of 2/m^2^. Using our model predictions, we find that inside the MPAs 11 “large‐sized” individuals (average TL 38.2 cm) per 60‐m^2^ transect corresponds with 2 urchins/m^2^, compared with 91 “small‐sized” individuals per transect (average TL 24.6 cm) to result in that same urchin density. To obtain that same urchin density in the reference areas would require a density of 24 large‐sized or 210 small‐sized CA sheephead per transect.

The top model (lowest AICc and highest AICc weight [1.00]) for macroalgal density included effects of temperature, depth, and purple urchin densities (Appendix S1: Table S3a, Appendix S1: Fig. S4), showing a strong negative association between purple urchin density and macroalgal density.

## Discussion

We found that healthy kelp forest ecosystems harbored trophic redundancy in the urchin predator guild. Additionally, we found relationships between the observed size class distributions of certain predators and urchin populations. Populations that included large predators were more effective at controlling urchins. These differences in predator demographics were related to levels of protection across an MPA network (Lafferty 2004, Hamilton et al. 2010a *a*, Caselle et al. 2015, Selden et al. 2017). Using the NCI as a model ecosystem for kelp forest dynamics, we showed that the effects CA sheephead, CA spiny lobster, and sunflower sea stars had on purple urchin densities varied as a function of the presence of the other urchin predators, ultimately leading to the differences in the persistence of canopy forming giant kelp and understory macroalgae. In MPAs, prior to the disease outbreak, purple urchins were exposed to predation from a three‐pronged predator guild that effectively maintained low urchin densities. With the onset of the sea star disease and widespread extirpation of sunflower sea stars, the predation pressure by CA spiny lobsters and CA sheephead on purple urchins was still strong enough to maintain low purple urchin densities inside the MPAs allowing macroalgae to persist (Figs. 2 and 4). In addition to protection from fishing afforded by the MPAs, the loss of the sunflower sea star might also have benefited the other two predators by releasing them from competitive pressures. However, outside of the MPAs, these fished predators were incapable of compensating for the loss of sunflower sea stars, and urchin barrens became common. The greater predation pressures exerted by CA sheephead and CA spiny lobsters in MPAs are likely driven by the intact size structures and higher abundances resulting from protection from fishing (Hamilton et al. 2010a). However, the predictions from the models indicate that CA sheephead at the reference sites might exert weaker predation pressures on purple urchins than CA sheephead of the same size and abundance inside the MPA. Although this intriguing result requires further research, we hypothesize that CA sheephead may preferentially avoid eating urchins from barrens because of a lower quality and lack of gonads in barren urchins, as was found for CA spiny lobster by Eurich et al. (2014). This pattern may also be explained in part by the additive predation pressure resulting from higher densities of CA spiny lobster in the MPAs (Kay et al. 2012). One particularly interesting result of our modeling of relationships between different size classes of CA sheephead and urchin abundance was the large disparity in the density required to maintain urchins at low densities across size classes of CA sheephead. Both in and out of the MPAs, the number of small sheephead required is nearly an order of magnitude higher than the number of large sheephead required to maintain the same density of urchins. Given the difficulty for small predators to compensate for the functional role of large predators, this result further emphasizes the importance of MPAs in restoring size distributions of fished species. Furthermore, in addition to the direct consumptive effects of predators on urchin populations, higher densities and diversity of predators in MPAs can potentially magnify trophic cascades indirectly because of effects on prey behavior (Byrnes et al. 2006, Spyksma et al. 2017). It is also important to note that CA sheephead are diurnal predators and CA spiny lobster are nocturnal predators, resulting in temporal variation in predation risk which could also have impacts on urchin grazing behavior and degree of urchin predation. Lobsters may have been underestimated by our daytime, visual surveys, but clear MPA effects on their abundance and size have been shown in the NCI (Kay et al. 2012). Our study lends support to the effectiveness of MPAs not only for restoring the biomass of fished species (Hamilton et al. 2010a *a*), but also for enhancing ecosystem resilience through trophic redundancy and restoration of biodiversity and size class structure.

In southern California, there is evidence that kelp forest/urchin barren dynamics operate at finer spatial scales (<500 m) than in other sections of the Pacific coast, where physical forces such as wave disturbance have been shown to influence regional trends in urchin and kelp abundance (Dayton et al. 1984, Reed et al. 2011, Cavanaugh et al. 2013, Karatayev et al. 2019). Our findings are consistent with these fine‐scale patch dynamics in urchin and algal abundances in our study region (Appendix S1: Fig. S1). Along with Southern California’s finer‐scale feedbacks between grazers and kelps, we know that abiotic (physical and chemical) factors are important for structuring macroalgal communities (Dayton 1985, Reed et al. 2011, Cavanaugh et al. 2013) and likely contribute to some of the temporal variation documented in our data sets. However, the spatial configuration of our sites is appropriate for controlling those factors to evaluate the effects of the MPAs. Sites are closely paired, in and out of MPAs at three islands, thus we would not expect abiotic factors to be driving the differences we show here. Instead, though correlative, the MPA effects we document are more consistent with trophic effects.

The NCI are situated in a biogeographic transition zone where the cold waters of the California current meet the warmer southern California countercurrent (Harms and Winant 1998). The resulting strong east–west temperature gradient is associated with variation in kelp forest communities across the islands (Hamilton et al. 2010b *b*, Caselle et al. 2015). Nearby Point Conception is a well‐recognized biogeographic boundary and this region is near the northern range limits for both CA sheephead and CA spiny lobsters (Fig. 1a), which are both associated with the warmer waters south of Point Conception and the eastern NCI (Murray et al. 1980, Burton 1998, Pondella et al. 2015). Prior to extirpation, sunflower sea stars were associated with the cooler waters north of Point Conception and the western NCI (Bonaviri et al. 2017). The overlapping range limits of these predators demonstrates an interesting spatial complementarity that potentially balances the weaker predator pressures exerted from CA sheephead and CA spiny lobsters at the cooler islands. North of Point Conception, sunflower sea stars and sea otters were the primary urchin predators prior to the onset of sea star disease; however, the patchy distribution of sea otters along the northern coast of North America has left large sections of the coast without a dominant urchin predator following the loss of sunflower sea stars. In some of these regions, such as northern California, the loss of macroalgae in those areas has been extensive. This mosaic of urchin predator distributions across the coast of North America (Fig. 1a) sets up interesting and testable hypotheses regarding the relationships between predator redundancy and coastwide resilience of kelp forests, which, we recommend, should guide future work.

At Anacapa Island, the easternmost and warmest of the NCI, CA spiny lobsters and CA sheephead are the primary predators of sea urchins (Caselle et al. 2018), and densities of sunflower sea stars were historically low even prior to their extirpation (Bonaviri et al. 2017). The island is zoned with varying levels of protection, which include a no‐take marine reserve, a partial‐take marine conservation area (commercial and recreational take of CA spiny lobsters permitted), and areas fully open to fishing. Caselle et al. (2018) showed that CA sheephead and CA spiny lobsters have strong direct negative effects on urchin abundance and an indirect positive effect on native algal abundance through their predation on urchins. Urchin densities were lowest and kelp densities highest in the no‐take reserve, where both urchin predators are protected. The inverse was observed in the fished area where CA sheephead and CA spiny lobsters are in low abundance. In the partial‐take MPA, where only one predator is protected, urchins and kelp were at intermediate densities, demonstrating the complementary effects of multiple predators such as we showed here. These spatial patterns are an indication that trophic redundancy enhances resilience and may help prevent ecosystems from shifting to alternative states. It also reinforces the importance of ecosystem‐based management strategies such as MPAs, which preserve functional roles at high trophic levels, thus restoring and preserving ecosystem integrity.

Areas along the coast that lacked diverse predator guilds or redundancy in the trophic structure were affected disproportionately more from loss of predators because of the sea star disease (Schultz et al. 2016). Sea otters, where they were present, maintained the predatory niche breadth, through trophic redundancy, in the absence of sunflower sea stars (Burt et al. 2018). However, in our study this trophic redundancy was only observed in the MPAs, because the redundant predators (i.e., CA sheephead and CA spiny lobsters) are subject to fisheries. Sea otter populations are positively correlated with black abalone populations and negatively correlated with urchin populations along the central coast of California, where sea otters are locally present (Raimondi et al. 2015). The sea otters suppress urchin populations, preventing urchin barrens from forming and allowing kelp to persist. The presence of the kelp forests creates a favored environment for black abalone food sources, thus demonstrating how the presence of a keystone species in a healthy kelp forest can indirectly harbor more diversity. In northern California, the recreational red abalone fishery is valued in the tens of millions of dollars (Reid et al. 2016). The California Fish and Game Commission closed the abalone fishery in 2018 for a minimum of three seasons, citing negative effects on abalone populations due to “extreme environmental condition” which broadly included the loss of the only key urchin predator to disease, massive increases in urchin barrens, and widespread losses of kelp (Rogers‐Bennett and Catton 2019). This is an example of how cascading ecological effects can reverberate through lower trophic levels resulting in devastating environmental effects that require drastic management actions that ultimately have profound economic consequences. Such consequences could perhaps be minimized, however, by placing additional management focus on trophic redundancy when possible.

As conservation efforts work to enhance ecosystem stability and buffer against anthropogenic change, management that promotes trophic redundancy has the potential to be highly effective. In coral reef systems, trophic redundancy has been found to play an important role in macroalgal abatement. With protection, there was significantly more functional herbivore diversity and functional redundancy when compared to fished areas, with the highest functional diversity and functional redundancy found in the large roving browsers of turf and macroalgae (Micheli et al. 2014). Through protection, these grazers proliferated, thus keeping the reef in a coral‐dominated state due to the resiliency that increased diversity brings. Diversity of grazer species can maximize herbivory processes leading to increased coral recruitment due to a release from competition with algae (Lefcheck et al. 2019). However, protecting the diversity needed to promote resiliency can be especially challenging for wildlife managers in naturally lower diversity systems such as those that occur at higher latitudes (Pianka 1966). Lower natural diversity at high latitudes combined with human harvesting may present additional challenges in preventing ecosystems from shifting to alternative states once disturbed. The interlocking web of stability a predator guild creates allows for ecosystems to absorb more perturbation than single predator systems. Mathematical modeling has indicated that ecosystem stability increases when multiple weak predator–prey interactions occur when compared to one strong predator–prey interaction (McCann et al. 1998). The presence of only one predator in a system creates a scenario where a perturbation to the predator populations could drastically shift the community to an alternative state.

As ecosystems are becoming more and more disturbed through climate change and anthropogenic stressors, it is important that we understand the mechanisms that prevent community‐wide phase shifts and ecosystem attributes that enhance resiliency. Our study demonstrates the importance of trophic redundancy in stabilizing perturbed system, which lends support to the previous theories, models, and small‐scale experiments on trophic redundancy (Steneck et al. 2002, Byrnes et al. 2006, McCary et al. 2016, Sanders et al. 2018), and identifies practical management actions that help to stabilize a key marine ecosystem in the face of a large‐scale ecosystem shock. Now more than ever, we need to protect our ecosystems’ predator guilds in order to maintain the stability of our ecosystems and prevent phase shifts in hopes of combatting ecosystem stressors.
