# Supplementary material for: Trophic redundancy and predator size class structure drive differences in kelp forest ecosystem dynamics
Source: Ecology. 2020 Feb 28;101(5):e02993. doi: 10.1002/ecy.2993 (PMC7317486; doi:10.1002/ecy.2993)
Supplement: Supplementary file 1 [file ECY-101-e02993-s001.pdf]

Supporting Information. Eisaguirre, J.H., J.M. Eisaguirre, K. Davis, P.M. Carlson, S.D. Gaines, and J.E. Caselle. 2020. Trophic redundancy and predator size class structure drive differences in kelp forest ecosystem dynamics. Ecology.

Appendix S1

Table S1. Generalized linear mixed effects models of urchin density pre sunflower sea star *extirpation from the western northern Channel Islands ranked by* Akaike's information criterion corrected *(AICc)*

Variables: Temp=Temperature, Pyc= Sunflower sea star*, MPA=Marine Protected Area, SheepTL=CA sheephead Total Length, Sheepab=CA sheephead abundance*

Model	AICc	ΔAICc	AICc Weight
a)Temp+Depth+Pyc	607.22	0.00	0.51
b)Temp+Depth	610.56	2.95	0.35
c)Temp+ Depth+Pyc* Sheepab*MPA	610.84	3.23	0.08
d)Temp+Depth+Pyc*SheepTL* MPA	611.47	3.87	0.06
e)Temp+Depth+Pyc*Sheepab* SheepTL*MPA	621.98	14.47	0.00

Table S2. Generalized linear mixed effects models of urchin density post sunflower sea star *extirpation from the western northern Channel Islands ranked by* Akaike's information criterion corrected *(AICc)*

Variables: Temp=Temperature, MPA=Marine Protected Area, SheepTL=CA sheephead Total Length, Sheepab=CA sheephead abundance

Model	AICc	ΔAICc	AICc Weight
a)Temp+Depth+sheepab*sheepTL*MPA	1318.43	0.00	0.99
b) Temp+Depth+SheepTL*MPA	1327.86	9.44	0.01
c) Temp+Depth+sheepab*MPA	1354.07	35.64	0.00
d)Temp+Depth	1355.73	38.96	0.00

Table S3. Generalized linear mixed effects models of algae density from the western northern Channel Islands ranked by Akaike's information criterion corrected *(AICc)*

Variables: Temp=Temperature, Urch=Purple Urchin

Model	AICc	ΔAICc	AICc Weight
a)Temp+Depth+Urch	1826.21	0.00	1
b)Temp+Depth	1876.57	50.35	0

Table S4: Variance inflation factors for top model pre-extirpation of sunflower sea stars

Temp=Temperature, Pyc= Sunflower sea star

Variable	Variance Inflation Factor
Depth	1.026241
Temp	1.019650
Pyc	1.039287

Table S5: Variance inflation factors for top model post-extirpation of sunflower sea stars

Temp=Temperature, MPA=Marine Protected Area, SheepTL=CA sheephead Total Length,

Sheepab=CA sheephead abundance

Variable	Variance Inflation Factor
depth	1.014785
temp	1.062645
sheep_ab	2.214613
sheep_tl	2.320415
MPA	1.054830

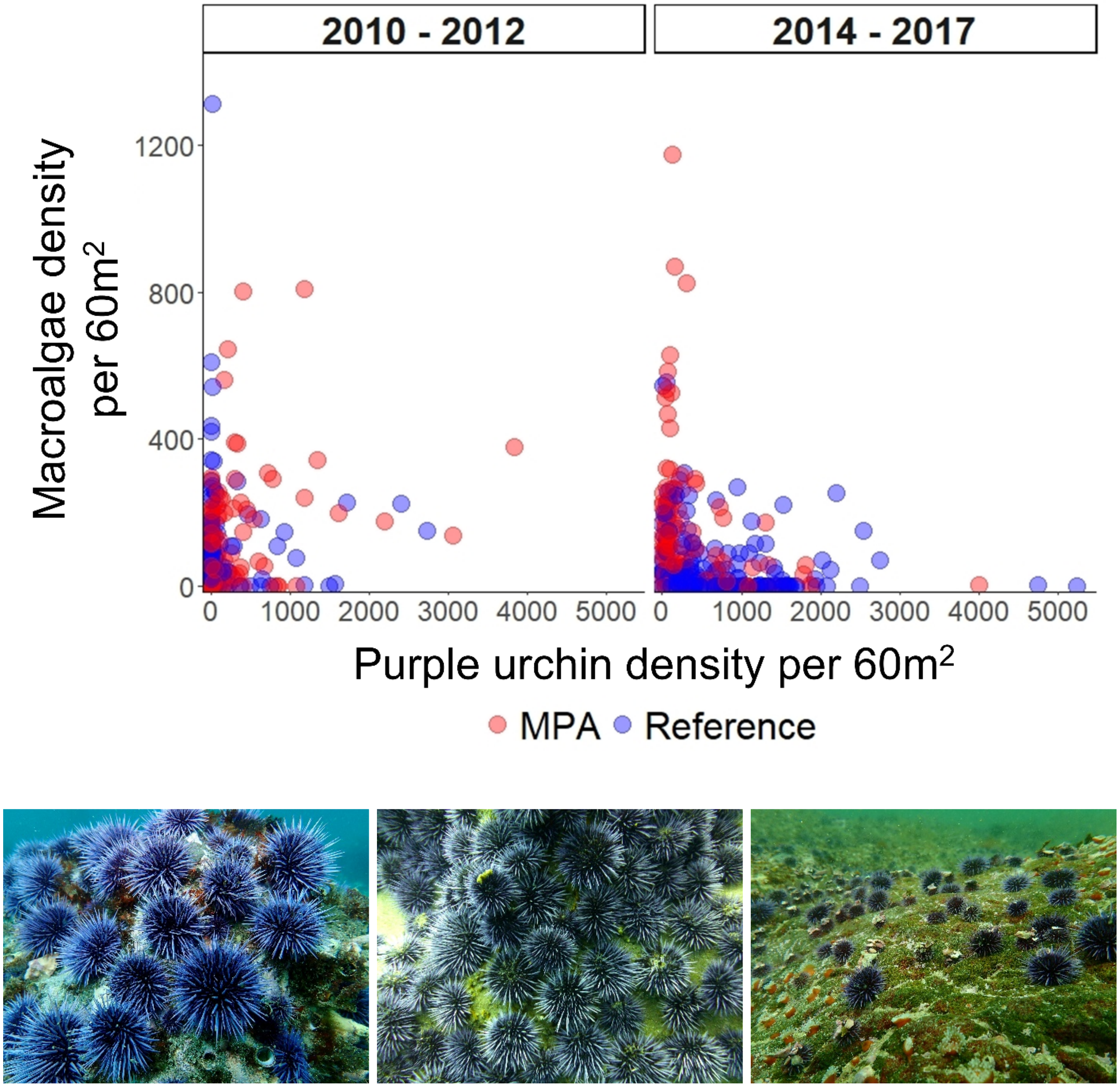

Figure S1. Raw, transect-level densities (no/60m^{-2}) of purple sea urchins and macroalgae (summed understory and giant kelp individuals) for pre-extirpation (2010-2012) *and post-extirpation (2014-2017 periods of sunflower sea stars. Bottom: Photographs of urchin barrens at several sampling sites in Reference areas at the western northern Channel Islands*

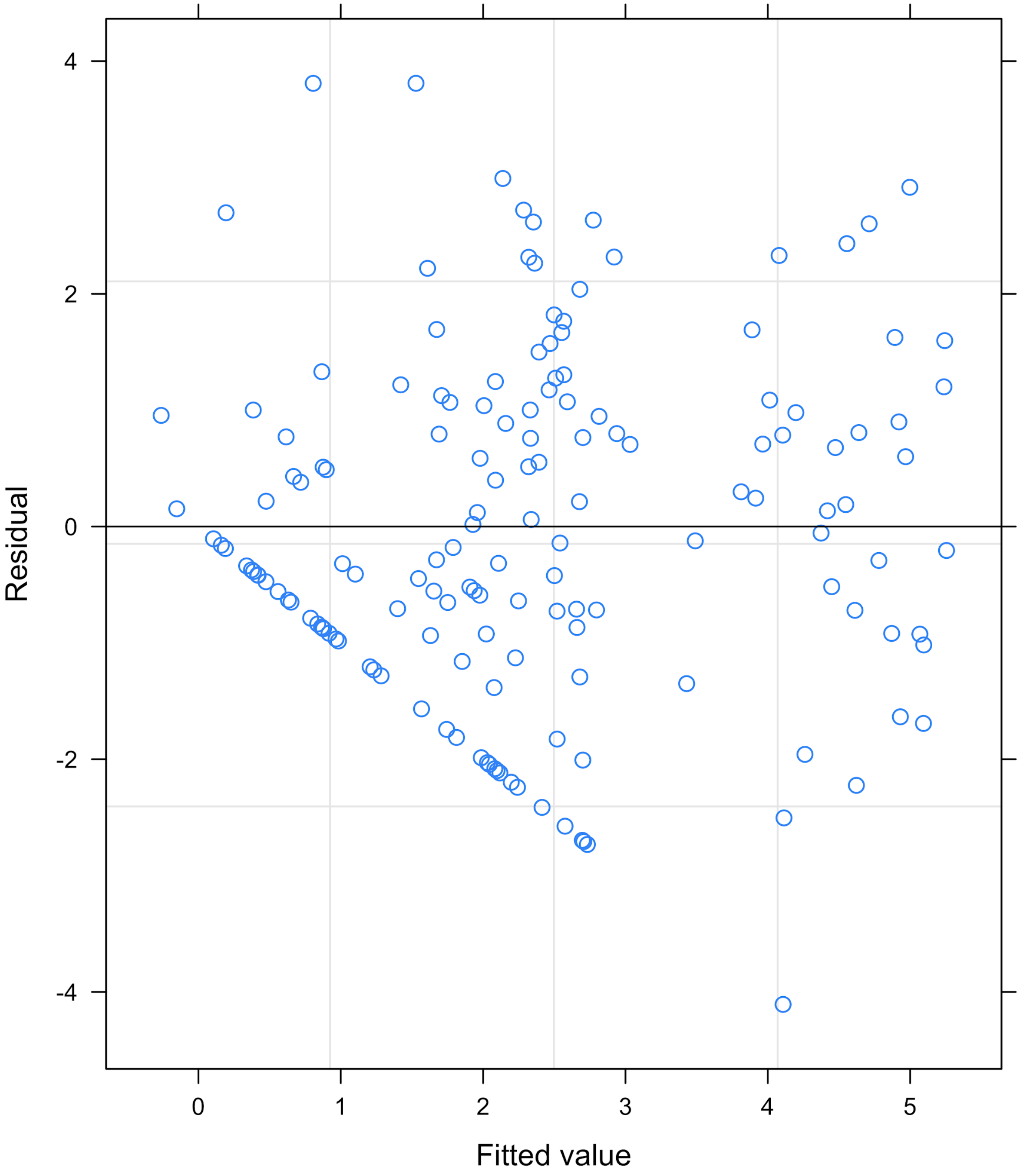

Figure S2. Residual plot for the top ranked models of urchin density pre-extirpation of sunflower sea stars

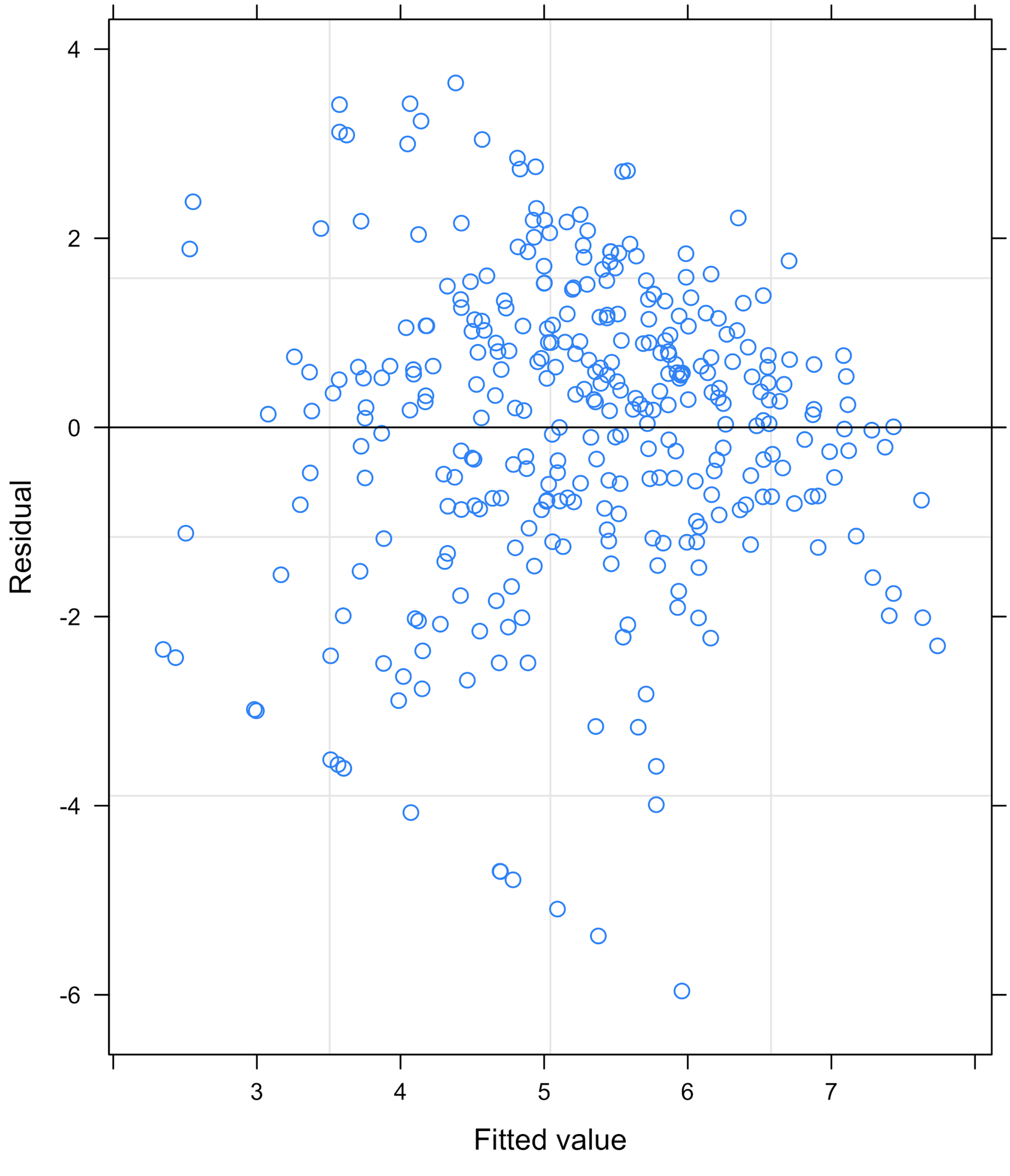

Figure S3. Residual plot for the top ranked models of urchin density post-extirpation of sunflower sea stars

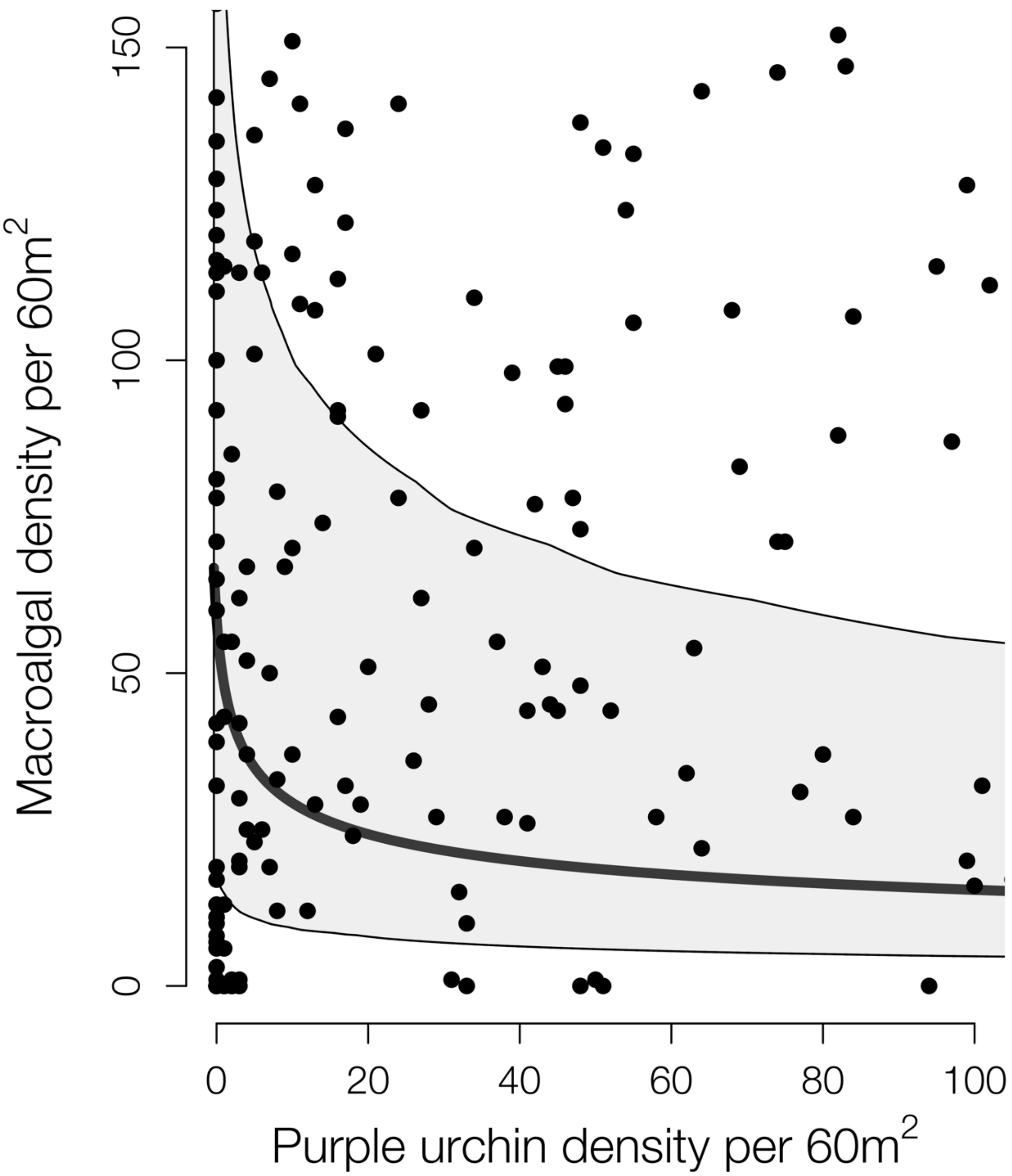

Figure S4. Empirical data (filled circles) and model prediction (line) of macroalgal densities (per 60 m^2) with temperature, depth, and purple urchin densities (per 60 m^2) as predictors with 95% prediction intervals at the western Northern Channel Islands.
